# Patient-Derived Fibroblasts With Presenilin-1 Mutations, That Model Aspects of Alzheimer’s Disease Pathology, Constitute a Potential Object for Early Diagnosis

**DOI:** 10.3389/fnagi.2022.921573

**Published:** 2022-07-01

**Authors:** Gustavo Lopez-Toledo, Maria-del-Carmen Silva-Lucero, Jorge Herrera-Díaz, David-Erasmo García, José-Antonio Arias-Montaño, Maria-del-Carmen Cardenas-Aguayo

**Affiliations:** ^1^Laboratory of Cellular Reprogramming, Departamento de Fisiología, Facultad de Medicina, Universidad Nacional Autónoma de México (UNAM), Mexico City, Mexico; ^2^Departamento de Fisiología, Biofísica y Neurociencias, Centro de Investigación y de Estudios Avanzados del Instituto Politécnico Nacional (Cinvestav-IPN), Mexico City, Mexico; ^3^Unidad de Servicios de Apoyo a la Investigación y a la Industria, Facultad de Química, Universidad Nacional Autónoma de México, Mexico City, Mexico; ^4^Departamento de Fisiología, Facultad de Medicina, Universidad Nacional Autónoma de México (UNAM), Mexico City, Mexico

**Keywords:** familial Alzheimer’s disease (FAD), fibroblasts, presenilin, neurodegeneration, post-translational modifications (PTM), autophagy, stress, proteomics

## Abstract

Alzheimer’s disease (AD), a neurodegenerative disorder that can occur in middle or old age, is characterized by memory loss, a continuous decline in thinking, behavioral and social skills that affect the ability of an individual to function independently. It is divided into sporadic and familial subtypes. Early-onset familial AD (FAD) is linked to mutations in genes coding for the amyloid-β protein precursor (*A*β*PP*), presenilin 1 (*PS1*), and presenilin 2 (*PS2*), which lead to alterations in AβPP processing, generation of the Amyloid-β peptide and hyperphosphorylation of tau protein. Identification of early biomarkers for AD diagnosis represents a challenge, and it has been suggested that molecular changes in neurodegenerative pathways identified in the brain of AD patients can be detected in peripheral non-neural cells derived from familial or sporadic AD patients. In the present study, we determined the protein expression, the proteomic and *in silico* characterization of skin fibroblasts from FAD patients with *PS1* mutations (M146L or A246E) or from healthy individuals. Our results shown that fibroblasts from AD patients had increased expression of the autophagy markers LC3II, LAMP2 and Cathepsin D, a significant increase in total GSK3, phosphorylated ERK1/2 (Thr^202^/Tyr^204^) and phosphorylated tau (Thr^231^, Ser^396^, and Ser^404^), but no difference in the phosphorylation of Akt (Ser^473^) or the α (Ser^21^) and β (Ser^9^) GSK3 isoforms, highlighting the relevant role of abnormal protein post-translational modifications in age-related neurodegenerative diseases, such as AD. Both 2-DE gels and mass spectrometry showed significant differences in the expression of the signaling pathways associated with protein folding and the autophagic pathway mediated by chaperones with the expression of HSPA5, HSPE1, HSPD1, HSP90AA1, and HSPE1 and reticular stress in the FAD samples. Furthermore, expression of the heat shock proteins HSP90 and HSP70 was significantly higher in the cells from AD patients as confirmed by Western blot. Taken together our results indicate that fibroblasts from patients with FAD-*PS1* present alterations in signaling pathways related to cellular stress, autophagy, lysosomes, and tau phosphorylation. Fibroblasts can therefore be useful in modeling pathways related to neurodegeneration, as well as for the identification of early AD biomarkers.

## Introduction

Alzheimer’s disease (AD) is the most common cause of dementia, accounting for approximately 60% of cases worldwide (Rizzi et al., 2014). The disease is histopathologically characterized by extracellular amyloid plaques composed of the Amyloid-β peptide (Aβ) and intracellular neurofibrillary tangles composed by the hyperphosphorylated tau protein. These two lesions have been associated with degeneration and neuronal death, synapse loss, and subsequent cerebral atrophy (Lane et al., 2018).

Alzheimer’s disease is classified into sporadic late-onset disease, representing more than 99% of cases, and early-onset familial disease, which represents 1% of cases. The histopathological hallmarks lesions of AD are neuritic plaques (NPs) and neurofibrillary tangles (NFTs). The most common type of Alzheimer’s disease usually begins after age 65 (late-onset Alzheimer’s disease, LOAD), which has been related to predisposing environmental factors such as diet, smoking, drinking alcohol in excess, low physical and mental activity, allelic predisposition by apolipoprotein E (APOE), and the triggering receptor expressed in myeloid cells; moreover, age is the main risk factor for developing AD. In contrast, familial AD (FAD) or early-onset AD (EOAD), begins between ages 30s and mid-60s. EOAD cases show autosomal dominant mutations in the AβPP, PS1, and PS2 (Masters et al., 2015).

Studies of FAD with mutations in AβPP, PS1, and PS2 support a causal link of the EOAD and the accumulation of abnormally folded Aβ and hyperphosphorylated tau protein in amyloid plaques and neuronal tangles. AβPP is the precursor of Aβ peptides, and mutations in the coding gene affect Aβ cleavage and aggregation. PS1 and PS2 are part of the catalytic subunit of the γ-secretase complex responsible, together with β-secretase, for AβPP cleavage, and mutations in PS1 or PS2 lead to less efficient AβPP processing and the generation of longer, hydrophobic peptides. The presence of neurofibrillary tangles composed of hyperphosphorylated tau is also required for AD diagnosis, and it has been proposed that tau pathological alterations follow Aβ deposition or that both proteins act in parallel in the development of the disease. However, mutations in the tau gene cause frontotemporal dementia without Aβ plaques, indicating that alterations on tau encoded protein induces neurodegeneration independently of Aβ (Iqbal et al., 2009; Scheltens et al., 2016). Thus tau post-translational modifications (PTMs) such as phosphorylations, acetylation, ubiquitination, glycation, glycosylation, SUMOylation, methylation, oxidation, and nitration, have a significant role in AD development, playing a critical role in tau localization, protein-protein interactions, maintenance of its levels, and modifying its aggregate structure (Alquezar et al., 2020). PTMs are essential to the normal function of tau and therefore alterations in the pattern of PTMs have the potential to lead to tau dysfunction, accumulation, and abnormal aggregation.

The diagnosis and treatment of neuropsychiatric disorders is hampered by the lack of precise information about the mechanisms involved in disease progression. Thus, models aimed at elucidating AD etiopathogenesis, identifying biomarkers, and developing therapeutic strategies are required. Some animal models (rat, mouse, dog, monkey, and primates) can mimic some aspects of human disease and metabolism; however, these models bear limitations due in part to the genetic differences between animals and humans, besides AD is a neurodegenerative disorder exclusive to the human being.

Nowadays, the only accurate AD diagnosis available is the postmortem, consistent in detection of NPs and NFTs in a brain slice. Clinical approaches to the presumptive AD diagnosis, including neuropsychiatric tests, neuroimaging, and the detection of proteins in cerebrospinal liquid, depend critically on the stage of the disease (Neugroschl and Wang, 2011). Therefore, the identification of peripheral biomarkers that allow for early diagnosis is essential to enable earlier interventions to slow disease progression.

Different studies have shown systemic molecular alterations in AD patients, consistent with those observed in the central nervous system. Some of these changes occurred in skin fibroblasts, lymphocytes, mesenchymal cells, platelets, and body fluids such as plasma and cerebrospinal liquid (Francois et al., 2014; Khan and Alkon, 2015; Delbarba et al., 2016; Trushina, 2019). In particular, fibroblasts from FAD patients show elevation in Aβ (Adler et al., 1991) and deficiencies in the autophagic flux, correlating with lysosomal abnormalities, dysfunctional mitochondrion accumulation (Martin-Maestro et al., 2017), errors in glucose metabolism and mitochondrial bioenergetics (Sims et al., 1985, 1987; Pérez et al., 2017), structural alterations and failure in signal transduction (Gibson et al., 1996; Zhao et al., 2002; Wang et al., 2008). These alterations are also observed in nerve cells derived from induced pluripotent stem cells (IPSCs), obtained from patient’s reprogrammed fibroblasts (Penney et al., 2020). This information indicates that cells such as fibroblasts can model pathological mechanisms characteristic of AD. Therefore, the study of peripheral cells could allow for identifying both biomarkers and therapeutic targets in AD. In this regard, differential expression analysis based on RNA-seq or proteomics in brain samples and peripheral cells could show differences in gene and protein profiles between peripheral cells from healthy individuals and FAD patients, in particular in molecules involved in crucial cellular processes related to neurodegeneration (Magini et al., 2010; Mukhamedyarov et al., 2016; Moradifard et al., 2018).

In the present study, we cultured fibroblasts from skin biopsies of *PS1* mutant FAD patients (FAD fibroblasts) and from apparently healthy individuals (control fibroblasts). All 12 cell lines (6 FAD and 6 controls) showed normal chromosomal number and expressed vimentin, a mesenchymal marker, and S100A4, a marker of this cell lineage [48, 49]. Fibroblasts from FAD patients with *PS1* mutations presented increased expression of the autophagy-lysosomal markers LC3II, LAMP2 and cathepsin D, increased total GSK3 levels and enhanced ERK1/2 (Thr^202^/Tyr^204^) and tau (Thr^231^, Ser^396^/Ser^404^) phosphorylation. Both 2-DE gels and mass spectrometry showed significant differences in the expression of the signaling pathways associated with protein folding and the autophagic pathway mediated by chaperones with the expression of HSPA5, HSPE1, HSPD1, HSP90AA1, and HSPE1 and reticular stress in the FAD samples. Furthermore, expression of the heat shock proteins HSP90 and HSP70 was significantly higher in the cells from AD patients as confirmed by Western blot. These data support the fact that somatic cells such as fibroblasts from FAD patients have distinctive protein expression and phosphorylation profiles that could serve as potential biomarkers and therapeutic targets.

## Materials and Methods

### Cell Culture

Skin fibroblast cultures were obtained from the Coriell Institute Cell Repository (Camden, NJ, United States). Cells were obtained from 6 *PS1* mutant AD patients from two different families (Italian, M146L mutation and Canadian, A246E mutation), or from 6 apparently healthy individuals from the same families (Table 1 and Supplementary Figures 1, 2). Fibroblasts cells from FAD patients were collected before the onset of AD symptoms.

**TABLE 1:** Characteristics of the fibroblast cell lines of individuals with familial AD (FAD-*PS1*) and control subjects.

Fibroblast population	*PS1* mutation	Catalog number (Coriell Institute)	Gender	Age	Position in the family tree
**Italian-FAD**					
AD1	M146L	AG08110	F	41	VI-2084
AD2	M146L	AG07872	M	53	V-249
AD3	M146L	AG08064	M	41	VI-2079
**Canadian-FAD**					
AD4	A246E	AG07629	M	54	VII-11
AD5	A246E	AG08170	M	56	VII-42
AD6	A246E	AG06840	M	56	VIII-64
**Italian-control**					
NA1		AG07936	F	63	V-236
NA2		AG08125	M	64	V-2100
NA3		AG08620	F	64	V-2078
**Canadian-control**					
NA4		AG07619	M	68	VI-40
NA5		AG07573	M	36	VIII-64
NA6		AG07621	M	57	VII-50

Fibroblasts were grown in minimal essential medium (MEM) with Earl salts (Gibco, Life Technologies, Grand Island, NY, United States) supplemented with 15% (v/v) non-inactivated Fetal Bovine Serum (FBS; ByProductos, Guadalajara, Jalisco, Mexico), 1% Glutamax (Gibco, Life Technologies), 1% penicillin-streptomycin (Gibco, Life Technologies), and 1% non-essential amino acids (*In vitro*, Mexico City, Mexico). Cells were maintained in an incubator at 37°C and 5% CO_2_/95% atmospheric air. The culture medium was changed every third day until cells reached 90% of confluence.

### Karyotype Analysis

Analysis was performed by Giemsa staining on each of the fibroblast lines. Cells were cultured in 25 cm^2^ flasks, and upon 90% confluence, the medium was replaced by a medium containing 0.1 μg/ml colcemid solution, and cultures were returned to the CO_2_ incubator. After 20 min, cells were collected using a 0.1% trypsin solution and suspended in 5 ml of a 75 mM KCl solution and incubated at 37°C for 20 min for nucleus obtention. One ml of ice-cold Carnoy’s fixative solution (methanol/acetic acid, 3:1) was added, and after gentle mixing, cells were centrifuged at 900 rpm for 10 min at room temperature. The supernatant was discarded, and after two additional fixation steps (resuspension in 5 ml of fixative solution and centrifugation at 900 rpm for 10 min), cells were resuspended in 200 μl ice-cold fixative solution, the suspension was dispersed on glass slides and incubated at 75°C for 3 h. Giemsa staining was then performed, and 20 karyotypes per slide were examined.

### Immunocytochemistry

Upon 90% confluence, cells were seeded in 24-well plates (200,000 cells/well) containing 12-mm diameter coverslips. After incubation for 24 h, cells were fixed with 4% paraformaldehyde for 20 min at room temperature and washed twice with 1× phosphate-buffered saline with glucose, GBS (5.4 mM KCl, 138 mM NaCl, 22 mM glucose, and 2 mM Na-KPO pH 7.2). Cells were then permeabilized with 0.2% Triton-X-100 in 1× GBS for 30 min at room temperature and incubated in blocking buffer [1% Bovine Serum Albumin (BSA) w/v, 0.2% Triton-X-100 v/v in 1× GBS]. After 60 min at room temperature, cells were incubated overnight at 4°C with primary antibody at the indicated dilution in blocking buffer. Cells were washed 3 times for 10 min with 0.2% Triton-X-100 in 1× GBS, and then incubated with Alexa Fluor^®^ 488-conjugated goat anti-rabbit IgG (H + L) secondary antibody (Thermo Fisher Scientific, Rockford, IL, United States; 1:500 dilution in blocking buffer) for 1 h at room temperature in the dark. Coverslips were washed 3 times with 1× GBS and nuclei were stained with DAPI (10 μg/ml) for 15 min before washing 3 times with 1× GBS. Finally, coverslips were mounted using Fluorogel (Electron Microscopy Sciences, Hatfield, PA, United States).

The antibodies tested were rabbit polyclonal anti-vimentin (1:250 from Santa Cruz, Dallas, TX, United States) and rabbit polyclonal anti-S100A4 (1:250 from Genetex, Irvine, CA, United States) (Table 2). The mounted coverslips were examined using a 40× oil immersion objective with a Leica TCS SP8 microscope (Leica, Wetzlar, Germany). Images were constructed using LAS X software version 3.7.1 (Leica, Wetzlar, Germany) and ImageJ software version 1.52p (NIH) with the FIJI image processing package (Schindelin et al., 2012).

**TABLE 2:** Antibodies used in immunofluorescence and western blot protocols.

Antibody	Catalog	Species	Description	Dilution	Company
Vimentin	Sc-6260	Rabbit polyclonal	Mesenchymal marker	1/250	Santa Cruz, Dallas, TX, United States
S100A4	GTX134743	Rabbit polyclonal	Fibroblastic marker	1/250	Genetex, Irvine, CA, United States
SQSTM1/p62	BS-2951R-TR	Rabbit polyclonal	Autophagy marker	1/1000	Bioss Antibodies Inc., Woburn, MA, United States
LC3	GTX17380	Rabbit polyclonal	Autophagy marker	1/1000	Genetex, Irvine, CA, United States
LAMP2	ABL-93-S	Rat monoclonal	Lysosomal marker	1/500	Developmental Studies Hybridoma Bank, Iowa city, IA, United States
CatD	GTX62063	Rabbit polyclonal	Lysosomal marker	1/1000	Genetex, Irvine, CA, United States
PSMB5	GTX50128	Rabbit polyclonal	Proteasome marker	1/1000	Genetex, Irvine, CA, United States
p-AKT Ser473	GTX50128	Rabbit polyclonal	Cell survival marker	1/1000	Genetex, Irvine, CA, United States
AKT	2938s	Rabbit polyclonal	Cell survival marker	1/1000	Cell Signaling Technology, Danvers, MA, United States
p-GSK3A/B	9327s	Rabbit polyclonal	Kinase marker	1/1000	Cell Signaling Technology, Danvers, MA, United States
total GSK3A/B	5676p	Rabbit polyclonal	Kinase marker	1/1000	Cell Signaling Technology, Danvers, MA, United States
p-ERK1/2	4695s	Rabbit polyclonal	Kinase marker	1/1000	Cell Signaling Technology, Danvers, MA, United States
total ERK1/2	9101s	Rabbit polyclonal	Kinase marker	1/1000	Cell Signaling Technology, Danvers, MA, United States
p-tau Thr^231^	MBS9600919	Rabbit polyclonal	Tau phosphorylation marker	1/1000	Biosource, San Diego, CA, United States
p-tau Thr^181^	GTX50171	Rabbit polyclonal	Tau phosphorylation marker	1/1000	Genetex, Irvine, CA, United States
p-tau Ser^396^/Ser^404^ (PHF-1)	ab184951	Rabbit polyclonal	Tau phosphorylation marker	1/1000	ABCAM, Cambridge, MA, United States
p-tau Ser^202^/Ser^205^ (AT8)	MN1020	Mouse monoclonal	Tau phosphorylation marker	1/1000	Thermo Fisher Scientific, Waltham, MA, United States
total tau (tau5)	556319	Rabbit polyclonal	Tau phosphorylation marker	1/1000	BD Pharmingen Inc., San Diego, CA, United States
AβPP (6E10)	SIG-39320	Rabbit polyclonal	Tau phosphorylation marker	1/1000	BioLegend, San Diego, CA, United States
HSP70	GTX104126	Rabbit polyclonal	Stress cellular marker	1/1000	Genetex, Irvine, CA, United States
HSP60	MCA-1C7	Mouse monoclonal	Stress cellular marker	1/1000	EnCor Biotechnology, Gainesville, FL, United States
GAPDH	GTX627408	Mouse monoclonal	Load control	1/1000	Genetex, Irvine, CA, United States

### *In silico* Analysis

Based on the gene expression analysis performed by Antonell et al. (2013) on 21 brain samples (thalamus level) from individuals with FAD and controls, we analyzed in the Gene Expression Omnibus (GEO) database GEO2R algorithm data from 7 control individuals and 7 FAD *PS1* mutant patients (3 with the M139T mutation, 2 with the V89L mutation, and 1 with the E120G mutation) aged 45–64 years old. The distribution of the differential expression data of the selected samples was determined using the value distribution command, and data from the comparison for the expression of 3,400 genes between the control and the FAD groups were exported to Excel format for further analysis and plotting.

### Western Blot

The 12 fibroblast cell lines from AD patients and controls were cultivated to 90% confluence. Cells were washed 3 times with 1× GBS and then lysed with ice-cold RIPA buffer (PBS, 1% w/v NP-40 from Thermo Scientific, Rockford, IL, United States, 0.1% w/v SDS from Sigma-Aldrich, St. Louis, MO, United States, and 0.5% w/v sodium deoxycholate from Sigma-Aldrich, St. Louis, MO, United States), containing protease inhibitors (0.24 mg/ml AEBSF from Sigma-Aldrich, St. Louis, MO, United States, 8 μg/ml aprotinin from Sigma-Aldrich, St. Louis, MO, United States, 10 μg/ml leupeptin from Peptides International, United States, 4 μg/ml pepstatin Peptides International, United States, 5 mM benzamidine from Sigma-Aldrich, St. Louis, MO, United States) and phosphatase inhibitors (20 mM β-glycerophosphate from Sigma-Aldrich, St. Louis, MO, United States, 10 mM NaF, from Sigma-Aldrich, St. Louis, MO, United States, 1 mM Na_3_VO_4_ from Thermo Scientific, Rockford, IL, United States) plus 1 mM EDTA from Sigma-Aldrich, St. Louis, MO, United States and 1 mM EGTA Sigma-Aldrich, St. Louis, MO, United States. Protein extracts were frozen at −20°C overnight, then centrifuged at 14,000 rpm for 15 min at 4°C, and the supernatant was recovered. Total protein was quantified using the Pierce BCA Protein Assay Kit^®^ (Thermo Scientific, Rockford, IL, United States), following the manufacturer’s instructions. Proteins were separated on 10% or 12% SDS-PAGE gels and transferred to 0.22 μm nitrocellulose membrane Protran 0.45 μm from GE Healthcare, United States. Membranes were blocked in 5% w/v Non-fat dry milk (Bio-Rad, Hercules, CA, United States) in TBST solution (0.05% Tween 20, from Sigma-Aldrich, St. Louis, MO, United States, in TBS) at room temperature, before incubation (4°C, overnight) with primary antibodies diluted in blocking buffer. Membranes were then washed 3 times in TBST at room temperature, and subsequently incubated with secondary antibodies, namely peroxidase-conjugated anti-mouse or anti-rabbit IgG (Jackson ImmunoResearch Laboratories, West Grove, PA, United States), diluted in TBST. After washing 3 times with TBST, bands were visualized with chemiluminescent reagent, ECL (Immobilon Chemiluminescent HRP High Sensitivity Substrate, from Sigma-Aldrich, St. Louis, MO, United States), scanned and analyzed using ImageJ-FIJI software (NIH version 1.52p) and Image Lab software (Bio-Rad, Hercules, CA, United States). The primary antibodies used are listed in Table 2.

### Protein Extraction for Two-Dimensional Gel Electrophoresis (2-DE)

All reagents were purchased from Millipore Sigma (St. Louis, MO, United States), unless otherwise stated. Protein extraction was performed according to previous reports by Hajduch et al. (2005) and Herrera-Diaz et al. (2018), with some modifications. Two control fibroblast lines (NA2 and NA3) and two FAD fibroblast lines (AD1 and AD2) with the M146L mutation (Table 1), each fibroblast line was cultured in 9 cell culture flasks of 300 cm^2^. Upon reaching 95% confluence, cells were washed 3 times with 1× GBS and scrapped in 15 ml 1× GBS. The cell suspension was then centrifuged (1,000 rpm, 5 min) and the pellet was suspended in 10 ml buffer solution containing 50% (v/v) phenol (pH 8.8), and 50% 0.1 M Tris-HCl (pH 8.8), 0.9 M sucrose, 10 mM EDTA, 0.4% 2-betamercaptoethanol and EDTA-free protease inhibitors (Complete™, Roche Molecular Diagnostics, Pleasanton, CA, United States). Samples were homogenized with an Ultra-turrax T-25 homogenizer (IKA Works, Wilmington, DE, United States), kept on ice for 5 min, and centrifuged at 4,000 rpm for 40 min. The organic phase was collected, 25 ml of 0.1 M ammonium acetate in 100% methanol were added, and the mixture was allowed to stand at −20°C for 16 h for protein precipitation. Samples were centrifuged at 4,000 × *g* for 30 min, and the pellet was washed with 5 ml of 0.1 M ammonium acetate in 100% methanol (twice), 0.1 M acetone 80% (twice) and 70% ethanol (once). The pellet was dried at room temperature and then dissolved in 400 μl of isoelectric focusing (IEF) buffer containing 2 M thiourea, 8 M urea, 2% (v/v) Triton X-100, 0.05 M dithiothreitol (DTT), 0.4% (w/v) CHAPS and EDTA-free Complete™ protease inhibitors (Roche Molecular Diagnostics). After careful re-suspension, the mixture was centrifuged at 14,000 × *g* for 15 min to obtain a clear supernatant. The protein concentration was determined with Nanodrop (Thermo Scientific, Rockford, IL, United States) and protein integrity was analyzed by one-dimension SDS-PAGE at 12% and Coomassie staining for at least 16 h.

### 2-DE Gels and Analysis

Proteins were separated by two-dimensional electrophoresis according to the method described by Herrera-Diaz et al. (2018) with some modifications. Briefly, 350 μg of total protein extracted from 4 fibroblast lines (NA2, NA3, AD1, and AD2) were independently applied onto an 11 cm polyacrylamide gel strip with a pH immobilizer gradient (IPG Bio-Rad) with pH range 3–10 for 10 min. at room temperature, in a rehydration tray (Immobiline DryStrip Reswelling Tray, Bio-Rad). Subsequently, the strips were covered with mineral oil and the tray was transferred to a PROTEAN i12 IEF Cell isoelectric focusing unit (Bio-Rad) for active rehydration (12 h, at 20°C). Rehydration was immediately followed by five focusing steps: 500 V for 30 min (fast), 1,000 V for 1 h (gradient), 3,000 V for 1 h (gradient), 5,000 V for 2 h (gradient) and 8,000 V (fast) for 3 h, up to a cumulative voltage of 30–35 kV. After isoelectric focusing, the IPG strips were removed from the tray and incubated with SDS equilibration buffer (1.5 M Tris-HCl pH 8.8, 6 M urea, 30% v/v glycerol 5%, w/v SDS and 2% DTT) for 15 min, with stirring at room temperature, repeated twice and then incubated with SDS alkylation buffer (Tris-HCl 1.5 M pH 8.8, 6 M urea, 30% v/v glycerol, 5% w/v SDS and 2.5% iodoacetamide) for 15 min, with stirring at room temperature, repeated twice, and finally washed briefly in 1× SDS-PAGE running buffer. The washed strips were subjected to second dimension separation on denaturing 12% acrylamide gels in a SE 600 Ruby System (GE Healthcare) at 25°C with constant voltage (50 V) for 22 h at room temperature. Gels were fixed with 50% methanol for 30 min and then stained with Coomassie Colloidal (20% [v/v] ethanol, 1.6% [v/v] phosphoric acid, 8% [w/v] ammonium sulfate, 0.08% [w/v] Coomassie Brilliant Blue G-250) for at least 16 h. Finally, the gels were washed with double-distilled water until achieving the appropriate contrast for spot identification. 2-DE gels and Western blots were scanned with the GS-900 Calibrated Densitometer (Bio-Rad). The scanned images of the 2-DE gels were analyzed using SameSpots v5.1 software (TotalLab, Newcastle upon Tyne, United Kingdom) in the single spot differential analysis study option (all reagents were from Sigma-Aldrich, St. Louis, MO, United States, unless otherwise indicated).

### Identification of Proteins by Mass Spectrometry

Samples were processed as described previously by Duarte Escalante et al. (2018) with some modifications. Briefly, total protein extracts from fibroblasts NA2, NA3, AD1, and AD3 were treated with a 0.05 M DTT solution as a reducing agent. After 45 min, 0.03 M iodoacetamide was added and the mixture was incubated for 2 h at room temperature in the dark. The samples were then washed 3 times with 0.1 M ammonium bicarbonate solution and dehydrated with 100% acetonitrile under vacuum. Digestion was performed by adding 30 μl of a modified porcine trypsin solution (20 ng/μl; Promega, Madison, WI, United States) in 0.05 M ammonium bicarbonate solution, followed by incubation for 24 h at 37°C.

The resulting peptides were extracted twice in 50% (v/v) acetonitrile, and 5% (v/v) formic acid for 30 min with sonication. The volume was reduced by evaporation in a vacuum centrifuge and adjusted to 20 μl with 1% (v/v) formic acid. Mass spectrometric analysis was performed with an integrated nano-LC-ESI MS/MS system: quadrupole/time of light, synapt G2 high-definition mass spectrometer (Waters Corporation, Milford, MA, United States) equipped with a NanoLockSpray ion source, coupled online to a nanoacquity UPLC system (Waters Corporation, Milford, MA, United States).

### Data Processing and Protein Identification

Data processing was performed with the global ProteinLynx version 2.4 server and software with a Protein Lynx Global Server (PLGS; Waters Corporation). PLGS scores with confidence >95% were accepted as correct. The UNIPROT database^1^ was searched and the peptides were matched with the theoretical peptides of the proteins reported for the samples.

### Bioinformatic Analysis of Protein Profiles Obtained From Mass Spectrometry (Panther and String Databases)

From the lists of proteins reported by mass spectrometry analysis of the FAD fibroblasts (AD1 and AD2) and control fibroblasts (NA2 and NA3), we selected those proteins with a 99% probability score (PLGS score) of being present in the samples represented as an “OK” value equals to 2 (Supplementary Tables 2, 3). Proteins were separated according to their differential expression in cells from control individuals or from FAD individuals (Supplementary Table 4). Protein interactomes were constructed (functional protein association networks) using the Multiple Protein by Name section of the String database^2^, the functional enrichment of the network based on the biological process was analyzed using the data of counting proteins in the network that share a particular GO term, the strength of the enrichment defined as the relationship between the amount of proteins observed and that expected in a random of the same size and the false discovery rate that describes the significance of the enrichment. In parallel, using the Panther database^3^ for gene ontology classification, in the Gene List Analysis section, the protein lists of both groups were analyzed, selecting the functional classification study of biological processes and the cell process subclassification in pie graph view.

### Statistical Analysis

Statistical analyses were conducted using GraphPad Prism 7.0 (GraphPad Software, La Jolla, CA, United States) to evaluate expression differences between the spots of the 2-DE gels of control and FAD fibroblasts detected using the SameSpot software (v5.1). Data are presented as mean ± standard error. For analysis involving multiple groups, one-way ANOVA with *post hoc* Fischer’s, Tukey’s, or Bonferroni’s test (as appropriate) was used. For all other comparisons, Student’s *t*-test was used. For all purposes, *p* ≤ 0.05 was considered as statistically significant. In our results section, we decided to include the fold change of the significant differential expressed proteins, only with the aim to highlight our principal findings in a didactical and illustrative way, but all the statistical analysis can be found in the figures of each experiment. Furthermore, each experiment has an internal duplicate and 3 experimental replicates, hence each densitometric analysis corresponds to all of these replicates, which validates our statistical analysis. All the figures are the representative images of each triplicate experiment and include the standard errors.

## Results

### Fibroblast Culture and Characterization

**FIGURE 1:**
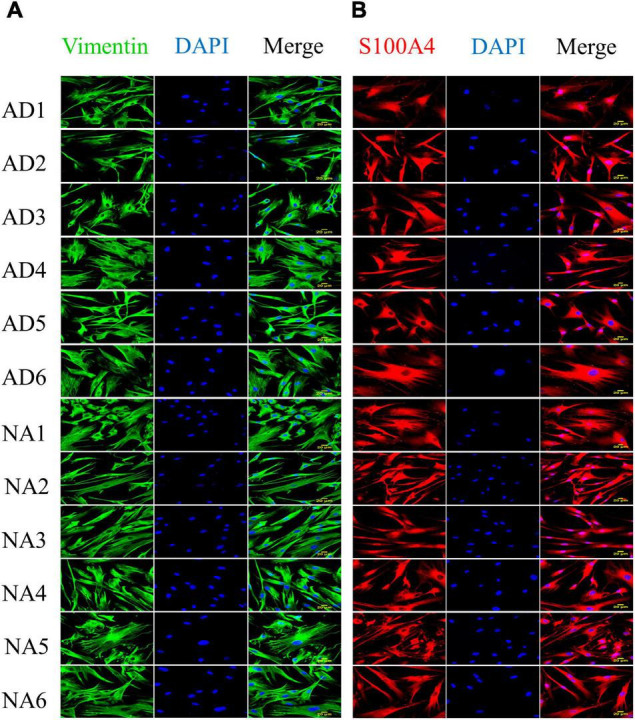
Expression of vimentin and S100A4 in fibroblasts with *PS1* mutations (AD) and fibroblasts without the mutation (NA). Protein expression was evaluated by immunocytofluorescence. **(A)** Expression of vimentin (green) in fibroblasts from control (6) and FAD (6) individuals. **(B)** Expression of S100A4 (red) in fibroblasts from control (6) and FAD (6) individuals. All cell lines show spindle and lamellar morphologies, and express both fibroblast cell markers. The images were obtained from culture passages 10–20, with a 40× objective and are representative of 3 determinations. Scale bars correspond to 20 μm.

The characteristics of the individuals with familial AD (FAD-*PS1*) and control subjects (gender, age, position in the family tree and *PS1* mutation when appropriate are specificized), as well as the catalog numbers from Coriell Institute of the corresponding fibroblast cell lines, are shown in Table 1. All 12 fibroblast lines from FAD (6) and control (6) individuals showed lamellar and fusiform morphology characteristic of the fibroblasts linage (Supplementary Figure 3), and proliferative capacity. Confocal microscopy indicated that all 12 cell lines expressed the protein Vimentin, a mesenchymal marker, and the protein S100A4, a marker of fibroblast cells (Figure 1).

The chromosomal stability of the fibroblasts from the FAD and control individuals was evaluated, and no aneuploidies were detected. Among the 12 cell lines, we identified 9 male (46; XY), and 3 female (46; XX) cell lines (Supplementary Figure 4). Of note, cells from the AD4 patient presented a translocation (17:21), previously reported by the Coriell Institute^4^.

### *In silico* Analysis of Gene Expression

Based on the gene expression data reported by Antonell et al. (2013) for 14 brain samples (from the posterior cingulate cortex, at the level of the thalamus) from 7 control individuals and 7 AD patients (M139T, V89L or E120G mutations in PSEN1), we detected significant differential gene expression (upregulation or downregulation) between FAD and control individuals in 3,400 genes (Supplementary Table 1).

We identify genes coding for membrane receptors or related to signaling pathways involved in cell survival, autophagy, proteasomal pathway, lipid metabolism, MAP kinases (MAPKs), inflammation, oxidative stress, tau protein phosphorylation, and genes associated with neurodegeneration (Table 3). We then analyzed the expression products of these genes by immunodetection in the fibroblast cell lines from AD patients and Controls.

**TABLE 3:** Genes associated with neurodegeneration in AD.

Gene name	Transcript ID	Fold change	Regulation	Description
**Autophagic-lysosomal**				
LAMP1	NM_005561	1.37	UP	Lysosomal-associated membrane protein 1
LAMP2	NM_013995	1.49	UP	Lysosomal-associated membrane protein 2
CATSH	NM_004390	2.19	UP	Cathepsin H
ULK1	NM_003565	−1.60	DOWN	Unc-51-like kinase 1
MAP1LC3A	NM_032514	−1.43	DOWN	Microtubule-associated protein 1 light chain 3α
**Proteasome**				
PSMB9	NM_002800	1.49	UP	Proteasome subunit, β type, 9
**MAP kinases**				
MAPK8	NM_002754	−1.37	DOWN	Mitogen-activated protein kinase 8
MAPK10	NM_002750	−1.71	DOWN	Mitogen-activated protein kinase 10
MAPK13	NM_138982	−2.11	DOWN	Mitogen-activated protein kinase 13
**Phosphorylation of tau protein**				
GSK3B	NM_002093	−1.41	DOWN	Glycogen synthase kinase 3β

### Analysis by Western Blot of Protein Expression

**FIGURE 2:**
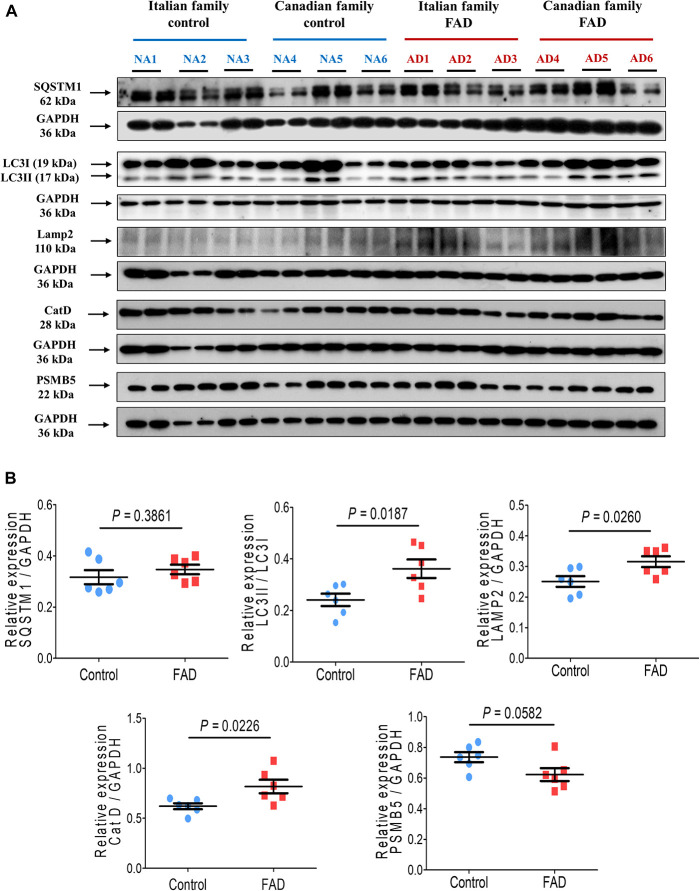
Immunodetection of autophagy (SQSTM1 and LC3II), lysosomal (LAMP2 and CatD) and proteasomal (PSMB5) markers in fibroblasts from FAD individuals and control subjects (NA). Proteins were evaluated by Western blotting. **(A)** Blots for fibroblast protein extracts from control (6) and FAD (6) individuals. Samples were analyzed in duplicate. **(B)** Analysis of SQSTM1, LC3II/LC3I, LAMP2, CatD and PSMB5. Protein levels were normalized to GADPH in the sample. The points on the graphs represent the average value of duplicates for each sample. Statistical analysis in **(B)** was performed with Student’s *t*-test.

Figure 2 shows the immunodetection of two autophagy markers, LC3II and SQSTM1. While there was no significant difference in SQSTM1 levels, the amount of LC3II was significantly higher (1.44-fold) in the fibroblasts of FAD patients cells as compared with cells from control individuals.

In regard to markers of the lysosomal pathway, there was a significant increase in the proteins LAMP2 and CatD in the fibroblasts from FAD patients (1.26- and 1.32-fold, respectively) in comparison with the control group (Figure 2). The proteasome pathway is an alternative to autophagic degradation pathway and we found no significant changes in the marker that corresponds to the active subunit 5 of the proteasome (PSMB5) in FAD fibroblasts (Figure 2).

**FIGURE 3:**
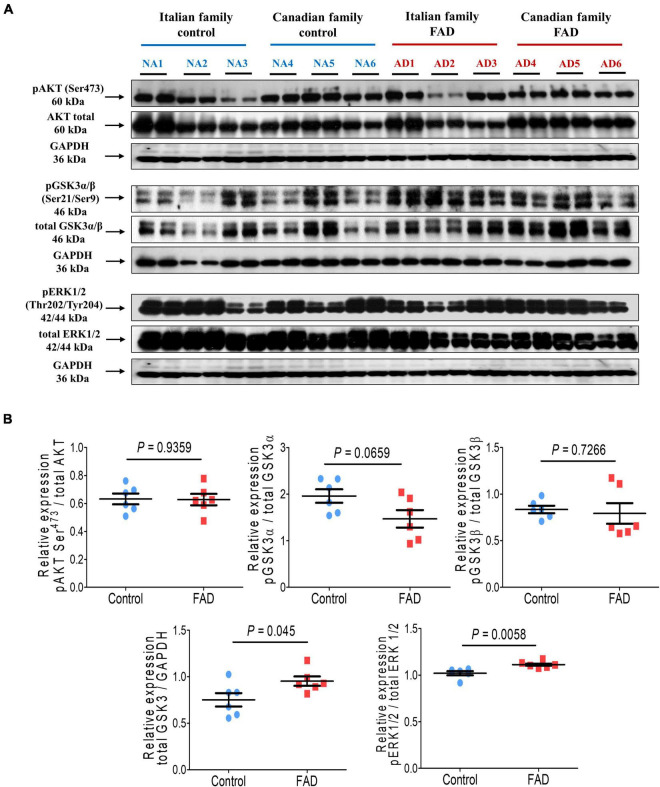
Immunodetection of cell survival markers (pAKT Ser^473^), phosphorylation of kinases involved in hyperphosphorylation of the tau protein (GSK3α Ser^21^, GSK3β Ser^9^, and ERK1/2 Thr^202^/Tyr^204^) in fibroblasts from FAD individuals and control subjects (NA). Proteins were evaluated by Western blotting. **(A)** Blots for fibroblast protein extracts from control (6) and FAD (6) individuals. Samples were analyzed in duplicate. **(B)** Analysis of pAKT Ser^473^, GSK3α Ser^21^, GSK3β Ser^9^, total GSK3 and ERK1/2 Thr^202^/Tyr^204^. Protein phosphorylation levels were normalized to their total protein content and total GSK3 was normalized against GAPDH. The points on the graphs represent the average value of duplicates for each sample. Statistical analysis in **(B)** was performed with Student’s *t*-test.

We then analyzed the Akt kinase (PKB) expression, which is related to cell survival, and found no significant difference in Akt phosphorylation at Ser^473^ (Figure 3). GSK3 is a critical downstream element of the PI3K/Akt cell survival pathway whose activity can be inhibited by Akt-mediated phosphorylation at Ser^21^ of GSK3α and Ser^9^ of GSK3β. Moreover, the kinases GSK3 and MAPKs can phosphorylate tau protein. No difference was observed in the phosphorylation of the GSK3β at residue Ser^9^ in the fibroblasts of FAD patients, although a trend toward reduced phosphorylation at Ser^21^ was observed for the GSK3α isoform, suggesting an increase in GSKα activity. Of note, total GSK3 protein levels were significantly higher (1.27-fold) in cells from FAD patients (Figure 3). As mentioned before, MAPKs are also involved in pathological tau phosphorylation, along with these fibroblasts from FAD patients showed a modest (1.09-fold) but significant increase in ERK1/2 phosphorylation at Thr^202^/Tyr^204^ (Figure 3).

**FIGURE 4:**
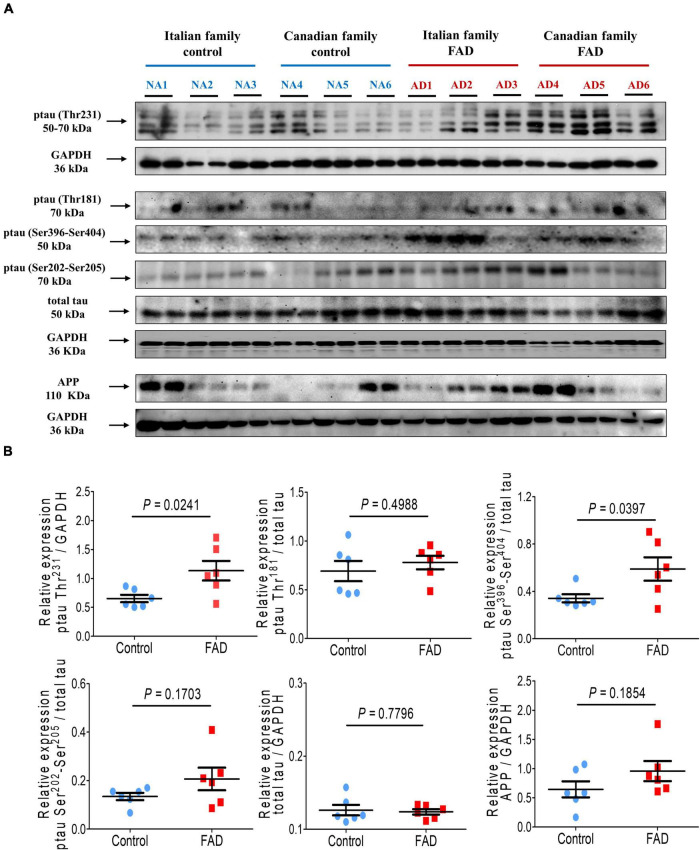
Immunodetection of the Amyloid β protein precursor (AβPP) and the pathological phosphorylation of the protein tau (Thr^231^, Thr^181^, Ser^404^, and Ser^396^) in fibroblasts from FAD individuals and control subjects (NA). Proteins were evaluated by Western blotting. **(A)** Blots for fibroblast protein extracts from control (6) and FAD (6) individuals. Samples were analyzed in duplicate. **(B)** Analysis of AβPP, p-tau-Thr^231^, p-tau-Thr^181^, p-tau Ser^396^-Ser^404^ and total tau levels. The amount of AβPP and total tau was normalized with GAPDH, and phosphorylation of tau protein was normalized with total tau levels. Points on the graphs represent the average value of the duplicates for each sample. Statistical analysis in **(B)** was performed with Student’s *t*-test.

In addition, tau phosphorylation at Thr^231^ and Ser^396^/Ser^404^, associated with AD pathology, was significantly increased (1.74- and 1.72-fold, respectively) in FAD patients fibroblasts. In contrast, phosphorylation at Thr^181^, Ser^202^/Ser^205^, and total tau expression were not different from control individuals (Figure 4), likewise we did not observe changes in the expression of AβPP between the fibroblasts of patients with a *PS1* mutation and controls (Figure 4).

### 2-DE Gel Analysis of Familial Alzheimer’s Disease and Control Fibroblasts

**FIGURE 5:**
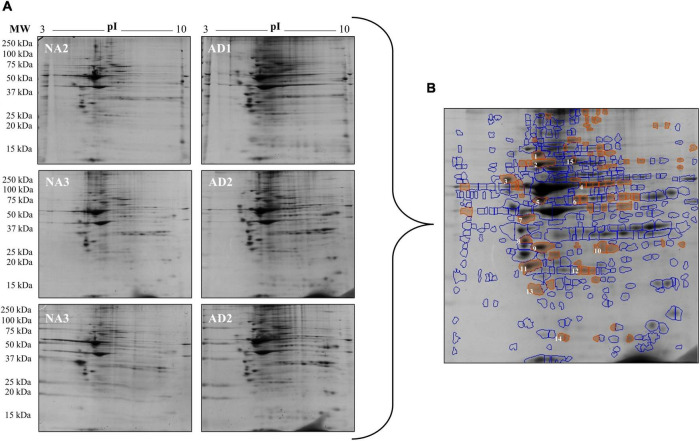
Changes in protein expression in fibroblasts from FAD patients (AD1 and AD2) and fibroblasts from control individuals (NA2 and NA3). Proteins were separated by molecular weight and isoelectric point using 2-DE gels. **(A)** Scanned images of the 2-DE gels developed with Coomassie blue; 2 biological replicates and 2 experimental replicas are presented. **(B)** Representative image of the 68 spots (red) with significant differences reported by SameSpot software, of which 15 proteins were identified by reference proteomic maps derived from fibroblasts. The analysis of expression is presented in Table 4.

The densitometric analysis of the spots on the 2-DE gels of the total protein extracts of FAD (AD1 and AD2) and control (NA2 and NA3) fibroblasts showed significant differences in 68 of 410 spots identified with SameSpots software v5.1 (Figure 5). Based on the coincidence of spots of the experimental gels with those of a reference map of fibroblastic proteins (Boraldi et al., 2003; Karring et al., 2004; Puricelli et al., 2006; Magini et al., 2010) with Isoelectric focusing (IEF) of pH-3-10, 15 differential proteins were identified as highly expressed in fibroblasts bearing a mutation in *PS1*, among which HSP90, HSP70, and HSP27 showed the highest significance and predominance (Table 4).

**TABLE 4:** Spots identified in the 2-DE gels of FAD fibroblasts and control.

Spot	Protein	pI-pH (experimental)	Mw kDa (experimental)	Average normalized volumes		Fold change	ANOVA (*P*)
				Controls	FAD		
1	HSP90	5.21	91	2.432e+007	5.138e+007	2.1	6.536e-004
2	HSP70	5.22	74	9.669e+007	1.449e+008	1.5	0.048
3	CRTC	4.43	62	2.749e+007	5.187e+007	1.9	0.031
4	TCPB	6.28	57	9.962e+006	2.503e+007	2.5	0.044
5	ATPB/PDA6	5.28	48	3.255e+007	5.442e+007	1.7	0.036
6	ENOA	6.19	48	1.062e+007	2.279e+007	2.1	0.003
7	SET	4.86	40	3.114e+007	5.682e+007	1.8	0.026
8	TPM1	4.87	33	2.722e+007	4.812e+007	1.8	0.032
9	ANXA	5.23	31	7.722e+007	1.065e+008	1.4	0.021
10	ESTD	6.75	31	1.011e+007	1.891e+007	1.9	0.023
11	CANS	4.97	27	5.690e+007	1.150e+008	2.0	0.017
12	HSP27	6.28	26	3.624e+006	6.479e+006	1.8	0.008
13	ATPQ	5.22	22	1.761e+007	2.624e+007	1.5	0.035
14	FINC	5.87	16	1.947e+006	4.673e+00	2.4	0.002
15	CALD	6.07	80	9.831e+006	1.592e+007	1.6	0.031

*Volumes were calculated from the densitometry analysis and normalized to total volume in the corresponding gel using SameSpot v5.1. The statistical comparison was performed with ANOVA.*

### Proteomic Analysis of Familial Alzheimer’s Disease and Control Fibroblasts

**FIGURE 6:**
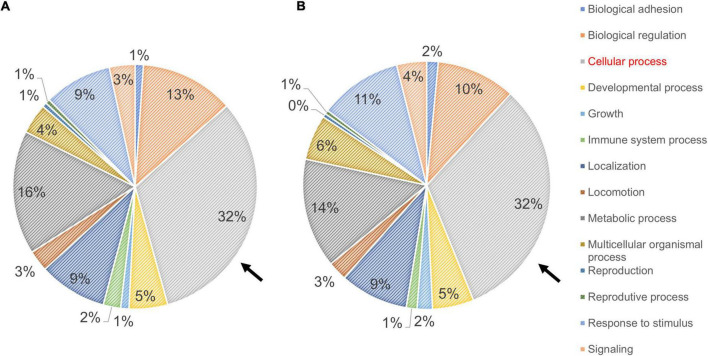
Ontological classification of the molecular function of proteins identified in fibroblasts derived from FAD individuals (AD1 and AD2) and apparently healthy individuals (NA2 and NA3). Mass spectrometry data derived from fibroblastic total protein extracts were analyzed in the Panther Gene ontology database. **(A,B)** Distribution of proteins from fibroblasts of the apparently healthy individuals **(A)** and FAD individuals **(B)** according to their molecular function. In both panels, values are expressed as percentage of the number of the proteins identified by mass spectrometry. The arrow indicates the classification of cellular processes selected based on their contribution to protein content among individuals.

**FIGURE 7:**
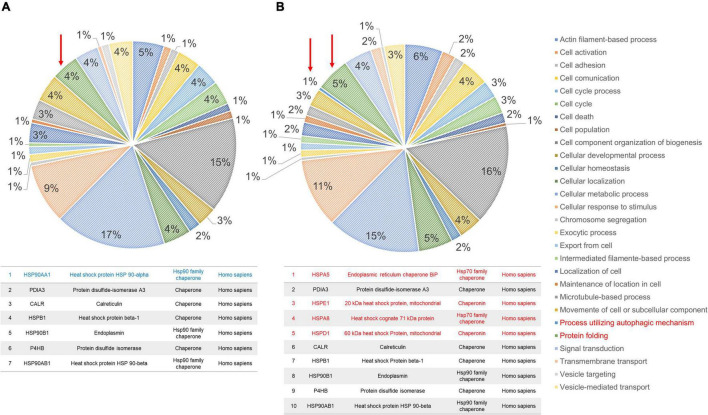
Ontological subclassification of cellular processes of the proteins identified in fibroblasts derived from patients (AD1 and AD2) and controls (NA2 and NA3). From the ontological classification of molecular function, the subclassification of cellular processes (accounting for ∼30% of the proteins identified by mass spectrometry) was selected. **(A)** Distribution of proteins from fibroblasts of the control individuals according to the cellular process in which they are involved. **(B)** Distribution of proteins from fibroblasts of the FAD individuals according to the cellular process in which they are involved. In both panels, values are expressed as percentage of the number of the proteins identified in the molecular classification. The arrow and the box in red indicate the classification of protein folding. As shown in Supplementary Tables 2, 3, control individuals (NA2 and NA3) have lower numbers of protein folding-associated cellular stress response proteins.

**FIGURE 8:**
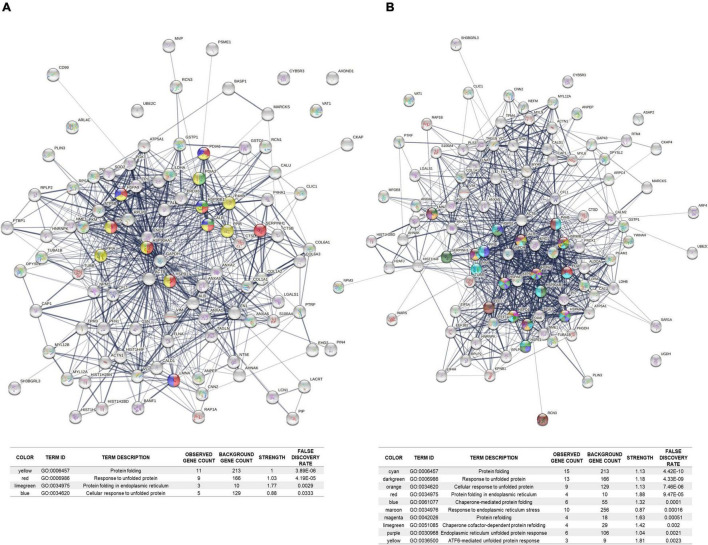
Cluster of stress response proteins (HSP) identified in the protein extracts of fibroblasts from the FAD patients (AD1 and AD2) with the mutation M146L. The proteins identified in the mass spectrometry of FAD fibroblasts (AD1 and AD2) and control fibroblasts (NA2 and NA3) were analyzed in the STRING database platform and interactomes based on functional and physical associations were generated. **(A)** Protein interactome for the fibroblasts of the control patients. **(B)** Protein interactome for the fibroblasts of the FAD patients (AD1 and AD2) identifying the presence of HSPs. For each interactome, the number of biological processes associated with protein folding mediated by chaperones is reported in the lower panel of this figure, both for the control group **(A)** and for the FAD group **(B)**. Pointing out the strength of the enrichment as a measure of what is observed and what is expected, and the value of the false discovery rate as a measure of the significance of the enrichment. The thickness of the lines between the proteins indicates the strength of the association.

The profiles obtained from mass spectrometry analyses (Supplementary Table 4) were evaluated in the Panther database to obtain genetic ontology charts classified by molecular function, subcellular location, and cellular processes. The samples from the FAD patients showed changes in classification, regulatory proteins, molecular function, subcellular localization, protein complexes, biological regulation, biogenesis, cellular process, locomotion, metabolic processes and signaling (Figure 6). From these data, the category of cellular processes was further analyzed given the greater distribution of proteins in both groups, identifying variations in the signaling pathways associated with protein folding and in the autophagic pathway mediated by chaperones with expression of HSPA5, HSPE1, HSPD1, HSP90AA1, and HSPE1 in the FAD sample (Figure 7). Mass spectrometry data were also submitted to the String Database platform to create interactomes, which showed a cluster of proteins associated with protein folding (Observed gene count = 25, Strength = 1.13, False discovery rate = 4.42E-10), response to unfolded protein (Observed gene count = 13, Strength = 1.18, False discovery rate = 4.33E-09), protein folding in endoplasmic reticulum (Observed gene count = 4, Strength = 1.88, False discovery rate = 9.47E-05), chaperone-mediated protein folding (Observed gene count = 6, Strength = 1.32, False discovery rate = 0.0001), response to endoplasmic reticulum stress (Observed gene count = 10, Strength = 0.87, False discovery rate = 0.00016), protein refolding (Observed gene count = 4, Strength = 1.63, False discovery rate = 0.00051), chaperone cofactor-dependent protein refolding (Observed gene count = 4, Strength = 1.42, False discovery rate = 0.002), endoplasmic reticulum unfolded protein response (Observed gene count = 6, Strength = 1.04, False discovery rate = 0.0021) and ATF6-mediated unfolded protein response (Observed gene count = 3, Strength = 1.81, False discovery rate = 0.0023) in the samples from the FAD individuals (AD1 and AD2) (Figure 8).

### Immunodetection of Heat Shock Proteins in Fibroblasts With PS1 Mutations

**FIGURE 9:**
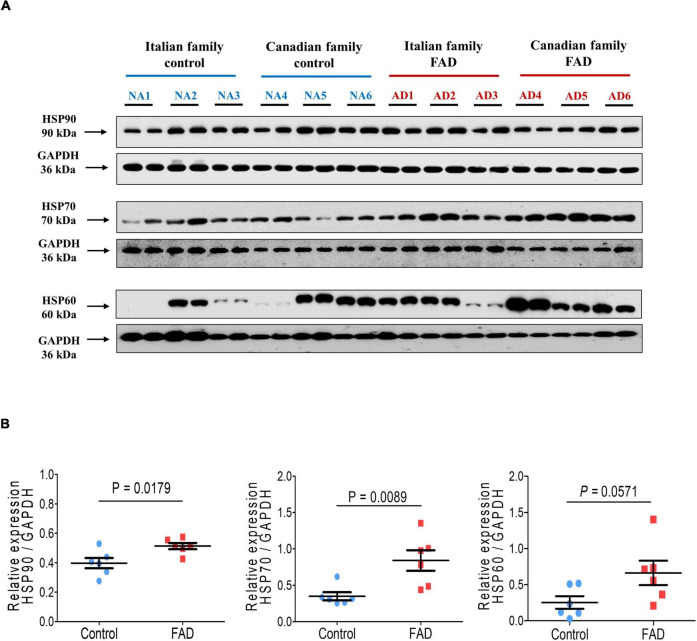
Immunodetection of the cellular stress markers HSP90, HSP70, and HSP60 in fibroblasts from FAD individuals and control subjects (NA). Proteins were evaluated by Western blotting. **(A)** Blots for fibroblast protein extracts from control (6) and FAD (6) individuals. Samples were analyzed in duplicate. **(B)** Analysis of HSP90, HSP70, and HSP60. Protein levels were normalized against GAPDH. The points on the graphs represent the average value of the duplicates for each sample. The statistical analysis was performed with Student’s *t*-test.

The proteomic analysis indicated differences at the molecular level between control individuals (NA2 and NA3) and FAD patients (AD1 and AD2), and protein expression of HSP90, HSP70, and HSP60 was therefore evaluated by immunodetection in the 12 fibroblast cell lines to validate alterations in these proteins related to cellular stress. HSP90 and HSP70 expression was significantly higher in the group with *PS1* mutations in comparison with the control group (1.29-and 2.42-fold, respectively), with a trend to increased HSP60 expression (Figure 9). These results support the findings of the analysis of the 2-DE gels and mass spectrometry, indicating an enhancing response to stress in FAD fibroblasts, likely due to the mutations in *PS1*.

## Discussion

Here we found that fibroblasts with mutations in *PS1* (A246E or M146L) present alterations at the level of the autophagic-lysosomal pathway, and increased expression or activity of kinases involved in tau phosphorylation. Moreover, we found that the expression of the heat shock proteins HSP90 and HSP70 was significantly higher in the cells from AD patients.

Changes in the lysosomal network proteins: the endosomal, lysosomal and autophagy systems, are among the first alterations observed in an AD brain (Nixon et al., 2005). In this study we found differential protein expression of major lysosomal and autophagy components. Autophagy flux is impaired in AD and in the brain and possibly in other organs/tissues of these patients. It has been reported an extensive autophagic vacuoles (AVs) accumulation within neuritic process and synaptic terminals, evidencing an impairment in the autophagy flux, either due to the hyper-induction of autophagy or to a reduction of AVs turnover. Thus, identifying alterations in the expression of components of the autophagy and lysosomal pathways in fibroblast cells could constitute an early biomarker of AD disease in peripheral tissues. In this respect, we found increased expression of the autophagy markers LC3II, LAMP2 and Cathepsin D in fibroblast cells from FAD patients. Microtube-associated protein light chain 3 (LC3II), a mammalian homolog of yeast Atg8, has been used as a specific marker to monitor autophagy. Upon induction of autophagy, LC3 is conjugated to phosphatidylethanolamine and targeted to autophagic membranes, thus an increased expression of LC3II represents and increased autophagy. Notwithstanding, it has to be taken into account that autophagy impairment in skin samples could not be an specific biomarker for AD, since autophagy is also impaired in skin diseases and other pathological conditions (Klapan et al., 2022); hence it has to be combined with other diagnostic criteria such as the detection of tau hyperphosphorylations and the clinical data.

Lysosomal-associated membrane proteins 1 and 2 (LAMP-1 and LAMP-2) are estimated to contribute to about 50% of all proteins of the lysosome membrane and to maintain the structural integrity of the lysosomal compartment. In embryonic fibroblasts, mutual disruption of both LAMPs is associated with an increased accumulation of autophagic vacuoles and unesterified cholesterol, while protein degradation rates are not affected (Eskelinen, 2006). It has been reported that the protein levels of LAMP-1 and LAMP-2 are increased in the CSF of AD patients, together with other four lysosomal network markers (LC3, Rab3, Rab7, and EEA1) (Armstrong et al., 2014). Armstrong et al. (2014) report that the increase in LAMP2 protein levels in AD patients CSF correlates with the well established early AD biomarker p-tau^181^ (tau phosphorylation at amino acid residue Thr^181^). This tau phosphorylation biomarker (p-tau^181^), together with the p-tau^217^ possess among other tau phosphorylation biomarkers the highest diagnostic power to discriminate between AD and non-AD dementias at an early stage (Struyfs et al., 2015; Palmqvist et al., 2020); thus LAMP2 elevation in CSF could be as useful as ptau^181^ and probably ptau^217^ for early AD diagnosis.

Thus our findings of increased autophagic-lysosomal pathway proteins, elevated expression of HSPs, augmented phosphorylation of kinases linked to increased tau phosphorylation observed in fibroblast cells could be associated to PS1 altered function, since mutations in PS1 gene are known to accelerate AD progression (Borchelt et al., 1997; De Strooper, 2007) by compromising the physiological functions of the proteins, such as AβPP intracellular traffic, which could lead to augmented AβPP processing and the subsequent increase in oligomeric forms and intracellular protein content, and potentially inducing tau pathology as reported for cellular and murine AD models (Gomez-Isla et al., 1997, 1999).

In this study, fibroblasts from FAD patients showed elevated expression of the markers LC3II, LAMP2 and CatD (Figure 2), likely reflecting the alterations in the autophagic lysosomal pathway described in neuronal cell models and transgenic mice with *PS1* mutations, due to the progressive accumulation of autophagic vacuoles and lysosomal deficiencies (Siman et al., 2000; Lee et al., 2010; Neely et al., 2011; Martin-Maestro et al., 2017). Lee et al. (2010) highlighted the role of PS1 in autophagy regulation. Autophagy, lysosomal acidification, and lysosomal proteolysis require PS1 and are disrupted by Alzheimer-related PS1 mutations, this is due to a selective impairment of autolysosome acidification and Cathepsin activation. Moreover, PS1 participates in the targeting of v-ATPase subunit to the lysosomes, which is essential in lysosomal acidification. In accordance with our results, this group also found that PS1 mutations causing early onset FAD, produce a similar lysosomal/autophagy phenotype in fibroblasts from AD patients as it occurs in neurons from AD patients. It is well known that PS1 cleaved form, known as the catalytic subunit of the gamma (γ)-secretase enzyme complex, is involved in the intramembranous cleavage of type 1 membrane proteins such as AβPP and Notch, but it has many other physiological functions. Some mutations on PS1 lead to increased accumulation of Aβ peptide, but different mutations affect other of its putative physiological functions such as cell adhesion, apoptosis, neurite outgrowth, calcium homeostasis, and synaptic plasticity and recently reported regulation of the autophagy-Lysosomal pathway (Shen and Kelleher, 2007). Although PS1 deletion affects autophagy proteolysis, it did not alter major aspects of macroautophagy, such as nutrient-dependent regulation of mTOR, generation of autophagosomes, evidenced by an increase in LC3II positive vesicles, degradation of p62 autophagy substrate and fusion of autophagosomes with lysosomes (which is independent of autophagolysosome acidification). Here we found that FAD fibroblasts with mutated PS1, have a clear increase in autophagosome formation, but most likely the degradation of its cargo is impaired (due to the lack of correct acidification).

The FAD is linked to mutations not only in PS1, but also in genes coding for *A*β*PP* and *PS2*, which lead to alterations in AβPP processing, generation of the Amyloid-β peptide and tau hyperphosphorylation. Accordingly, FAD animal models with mutations in *PS1* show molecular, biochemical and physiological modifications including memory alterations (Sun et al., 2005; Mirnics et al., 2008; Elder et al., 2010; Lee et al., 2010), reduced survival (Wen et al., 2004; Veeraraghavalu et al., 2013), impaired synaptic function (Priller et al., 2007; Auffret et al., 2009; Ho and Shen, 2011), alterations in AβPP processing, increased Aβ levels (Yu et al., 2001; Arber et al., 2020) and increased tau phosphorylation (Pigino et al., 2001; Shepherd et al., 2004; Tanemura et al., 2006; Ochalek et al., 2017), that altogether lead to neurodegeneration (Chui et al., 1999; Nixon et al., 2005; Eskelinen, 2006; Maarouf et al., 2008; Armstrong et al., 2014; Struyfs et al., 2015; Cacace et al., 2016; Palmqvist et al., 2020; Klapan et al., 2022).

Our FAD fibroblast cell lines came from two different families; thus, they have the following PS1 mutations: A246E and M146L, and Antonell et al. (2013), that published the work we use for the comparative bioinformatic analysis of differential gene expression, used brain samples of FAD patients with 3 different PS1 mutations: 1 M139T mutation, 2 with the V89L mutation, and 1 with the E120G mutation. Therefore, many authors study tissue or cell samples with different PS1 mutations (Piccini et al., 2004; Sproul et al., 2014; Martin-Maestro et al., 2017) because the main fact is the PS1 change in function due to its mutations, no the particular mutation, which among them they share the principal phenotype with little differences.

Autophagic dysfunction may be related to tau hyperphosphorylation as well as to protein truncation and oligomerization events, leading to alterations in the autophagic lysosomal flow, given the crucial role of the protein tau in autophagosome trafficking and maturation (Zheng et al., 2012; Liu et al., 2015). Furthermore, the lysosomal pathway is also critical for the metabolism of the Aβ peptide by eliminating autophagosomes containing the peptide, and increased LAMP2 and CatD levels have been observed in the latter process of AD pathology (Hoffman et al., 1998; Saido and Leissring, 2012; Sollvander et al., 2016; Whyte et al., 2017). As we mentioned before, in fibroblast cells from knockout mice with deletion of the *PS1* gene, lysosome-dependent proteolysis is compromised, with abnormally high levels of autophagy substrates such as p62 and LC3II (Lee et al., 2010; Martin-Maestro et al., 2017). Moreover, expression of the LC3II/LAMP2 markers in cells with *PS1* mutations indicates the presence of autophagosome-lysosome fusion, and potential Cathepsin release, because LAMP2 is required for lysosome maturation. Thus, PS1 is necessary for lysosomal acidification and final cargo degradation (Lee et al., 2010). Endocytic and lysosomal pathology is among the primary events of AD, and patients show alterations in the endolysosomal network in several brain regions before the presence of local damage induced by Aβ or tau aggregations (Penke et al., 2018). Here we found that levels of the complete form of AβPP were not altered in the FAD fibroblasts (Figure 4), as previously reported for neuronal models derived from reprogramming fibroblasts with a *PS1* mutation, although the βCTF form of AβPP increases in neurons with *PS1* mutations leading to increased expression of markers of the autophagic-lysosomal pathway. In this respect, several authors (Piccini et al., 2004; Sproul et al., 2014; Hung and Livesey, 2018) have evaluated the changes in AβPP processing in human neural cells reprogrammed from FAD PS1 mutated fibroblasts, but not specifically in fibroblast cells, therefore we will evaluate the potential presence of AβPP proteolytic products in patient derived fibroblasts, since this evaluation will contribute to validating the possible use of peripheral skin fibroblast cells in FAD diagnostic.

Taken together, the dysregulation of the expression of autophagic-lysosomal pathway key regulatory proteins in somatic cells, the presence of HSPs, the augmented phosphorylation of kinases linked to increased tau phosphorylation, as well as overexpression of AβPP, observed in the present work, could be associated with the PS1 mutations, because mutations in this gene are known to accelerate AD progression (Borchelt et al., 1997; De Strooper, 2007) by compromising the physiological functions of the protein, most likely in relation to AβPP intracellular traffic, which could lead to augmented AβPP processing and the subsequent increase in oligomeric forms and intracellular protein content, as reported previously for cells in culture and AD murine models (Gomez-Isla et al., 1997, 1999).

Our results highlight the critical role of abnormal protein PTMs in age-related neurodegenerative diseases, such as AD. The microtubule-binding protein tau can be phosphorylated, methylated, acetylated, glycosylated, nitrated, sumoylated, and truncated, among other PTMs. Tau aggregation and the generation of neurofibrillary tangles (NFTs), which are a hallmark of AD, are linked to PTMs such as hyperphosphorylation. Here we detected tau phosphorylations at residues Thr^231^, Ser^396^, and Ser^404^, which are important tau phosphorylations linked to AD. Tau phosphorylation at Thr^231^, together with tau phosphorylations at Thr^181^ and Thr^217^ are considered early biomarkers of AD, that raise their levels in CSF of AD patients, increasing early in the Alzheimer’s continuum, when only subtle changes in Aβ pathology are detected (Luna-Munoz et al., 2007; Suarez-Calvet et al., 2020). According to Augustinack et al. (2002), tau phosphorylation at Thr^181^ and Thr^231^, corresponds to a rectangle state of AD. Tau phosphorylation at Thr^231^ is located at the middle region of tau protein sequence, while phosphorylations at Ser^396^ and Ser^404^ are located at the C-terminal region of tau protein. Phosphorylation of tau protein at the carboxyl terminus may be among the middle to advance tau PTMs events, and it coincides with the generation of intracellular to extracellular neurofibrillary tangles (Augustinack et al., 2002; Mondragon-Rodriguez et al., 2014). Altogether, this result of the enhanced tau phosphorylation on tau residues Thr^231^, Ser^396^, and Ser^404^, confirms early to middle-advance tau PTMs probably associated to the initial steps to develop AD pathology, from the pre-NFT to the beginning of intracellular NFTs generation, since our cell model corresponds to fibroblast cells from FAD patients at a pre-symptomatic step (according to the data available at the Coriell Institute), and this could explain the absence of late-state markers of AD pathology in these cells. Furthermore, phosphorylations of tau protein in non-neuronal cells, such as fibroblasts, may be an indicative of the genesis of AD pathology that is nowadays been considered as a systemic disease instead of a central nervous system (CNS) exclusive disease (Kurakin and Bredesen, 2020). According to the systems biology analysis performed by Kurakin and Bredesen (2020), AD may not be a brain disease but a progressive system-level network disorder, which is driven by chronic network stress and dyshomeostasis in the whole organism. Independently if the chronic stress, toxicity, and inflammation originate in the brain or in the periphery, they are communicated with the (CNS) via humoral and neural routes, preferentially targeting high-centrality regulatory nodes and circuits of the nervous system, and eventually manifesting as a neurodegenerative CNS disease.

Disruption of Akt- (also known as PKB, protein kinase B) and ERK- (the extracellular signal-regulated kinases) mediated signal transduction significantly contributes to the pathogenesis of many neurodegenerative diseases, such as AD. These kinases regulate cell survival, motility, transcription, metabolism, and cell cycle progression. Therefore, ERK and Akt/PKB signaling pathways can be an effective therapeutic target to prevent the progression of neurodegeneration (Rai et al., 2019). ERK1/2, also known as p42/p44 or MAPK, plays vital role in neural function, participating in proliferation, differentiation, and survival. The MAPKs are serine/threonine protein kinases that promote a large diversity of cellular functions in many cell types. Three major mammalian MAPK subfamilies have been described: the extracellular signal-regulated kinases 1 and 2 (ERK1/2), the c-Jun NH2-terminal kinases (JNK), and the p38 kinases (Rai et al., 2019). Likewise, ERK1/2 and the Glycogen synthase kinase-3 (GSK3) (Figure 3), regulate the phosphorylation state of the microtubule-associated protein tau (Martin et al., 2013) and their overexpression or overactivation are among many of the pathological signs of the disease in sporadic and familial AD cases (Pei et al., 2002; Hooper et al., 2008; Giese, 2009). We cultured the fibroblasts from FAD in the presence of fetal bovine serum, we were able to detect an increase in ERK1/2 phosphorylation (Figure 3) as compared to control fibroblasts from Apparently Healthy Individuals. This ERK1/2 phosphorylation could be associated to *PS1* mutations, as reported for somatic cells from patients carrying *PS1* mutations (Zhao et al., 2002). Increased phospho-ERK has been found in brain extracts from AD patients (Russo et al., 2002), suggesting its role in the pathogenesis of AD. Furthermore, abnormal fibrillary depositions of proteins such as tau, α-synuclein and Aβ, that are characteristic of neurodegenerative disorders, are cytosolic targets of ERK’s, thus implicating MAPK pathway (chronic ERK1/2 activation) in formation/maintenance of such pathological hallmarks, and therefore in the noxious events that lead to the specific neurodegeneration. Moreover, prolonged activation of ERK could led to oxidative stress by ROS elevation (Simonian and Coyle, 1996). However, more research is required to clarify the mechanisms underlying the changes in ERK1/2 activity related to AD progression.

One of the downstream substrates of Akt is GSK3 among Bad, FOXOs and caspase-9 via Akt/PKB signaling pathways. GSK3 activation has been linked to the pathophysiology of a variety of neurodegenerative illnesses; therefore inhibition of this kinase has been proposed as a therapy option for these conditions (Hernandez et al., 2009). In mammals, GSK3 is encoded by two genes, gsk3α and gsk3β, expressing the proteins GSK3α (51 KDa) and GSK3β (47 KDa). Phosphorylation at Ser^21^(GSK3α) or Ser^9^ (GSK3β) could result in the inhibition of GSK3 activity. On the other hand, dephosphorylation of those phosphoserine residues could activate GSK3, Protein phosphatase 2A (PP2A) and protein phosphatase 1 (PP1) have been implicated in the control of these dephosphorylations (Planel et al., 2001; King et al., 2006). GSK3 is a kinase that plays an essential role in AD given its involvement in tau hyperphosphorylation, in our study, no difference was observed in the phosphorylation state of the GSK3β at residue Ser9 in the fibroblasts of FAD patients, although a trend toward reduced phosphorylation at Ser21 was observed for the GSK3α isoform, suggesting an increase in GSK3α activity; accordingly fibroblasts from FAD patients showed higher levels of total GSK3 (Figure 3). The increased expression/activity of GSK3 has been reported to be associated with memory impairment, increased Aβ production and inflammatory responses in AD (Singh et al., 1995; Grimes and Jope, 2001; Planel et al., 2001; Phiel et al., 2003). GSK3 also reduces acetylcholine synthesis, which is consistent with the cholinergic deficit characteristic of AD, and is a crucial mediator of apoptosis and may therefore contribute directly to neuronal loss (Giese, 2009). GSK3β interacts with PS1 heterodimeric complexes, and *PS1* mutations could thus dysregulate the molecular and functional interactions of PS1 and GSK3β affecting axonal transport and microtubule stability (Pigino et al., 2003). Moreover, PS1 can bring the protein tau and GSK3β closer together, allowing their interaction, along with *PS1* mutations present in FAD increase the protein capability to bind GSK3β; consequently, *PS1* mutants lead to enhanced tau phosphorylation (Takashima et al., 1998). It is possible that other GSK3 regulators could be involved in the overactivation of this enzyme in the FAD fibroblasts (in agreement with the overexpression of GSK3 observed here), since many GSK3 substrates require a previous (priming) phosphorylation by other kinase (priming kinase) at a residue located four amino acids toward the C-terminal of the residue to be modified by GSK3. Among these priming kinases are PKA, PKC, casein kinase I CDK5, or PAR1 (Hernandez et al., 2009); thus, theses kinases could regulate the phosphorylation of different substrates by GSK3. Besides GSK3 involvement in neurodegenerative disorders, it contributes to the development of several human disorders, like metabolic disorders, diabetes, and viral infections, among others; thus GSK3 inhibitors should correct the activity status of this enzyme.

As we mentioned, early stages of AD brain pathology are associated with tau protein phosphorylation at several amino acids residues such as Thr^231^ and Thr^181^, as well as with increased levels of PHF-1 and total tau (Figure 4) not only in brain cells but also in cells from peripheral tissues such as submandibular gland, sigmoid colon, liver, scalp and abdominal skin (Dugger et al., 2016), which supports our finding that FAD fibroblast showed enhanced tau phosphorylations at these epitopes, events that could be associated with *PS1* mutations that affect ERK1/2 and GSK3 expression or activity.

Many neurodegenerative diseases are thought to be caused by protein misfolding. Heat shock proteins (HSPs), which function mainly as molecular chaperones, play an important role in the folding and quality control of proteins, reducing the number and size of inclusions and accumulation of disease-causing proteins. Interestingly, we also found an increased expression of HSPs (HSP90 and HSP70) in FAD fibroblasts (Figures 5, 7, 8) which are involved in maintaining protein homeostasis through the regulation of protein folding and turnover via the proteasomal or autophagic pathways, which hold relevance in AD due to the accumulation of Aβ peptide and tau protein (Dou et al., 2003; Lu et al., 2014; Ou et al., 2014; Campanella et al., 2018). The affinity of tau protein for some molecular chaperones is greatly increased as a result of its conformational alterations. Hsp90 is a key cellular chaperone that forms huge complexes with several co-chaperones. Hsp90 complexes play a key role in protein quality regulation and protein breakdown via the proteasomal and autophagic-lysosomal pathways. These Hsp90 complexes have tau protein as a client protein. If tau protein is aberrant or mutated, it can cause CHIP protein (carboxyl terminus of Hsc70-interacting protein), an E3-activated co-chaperone, to be recruited to the complex, causing tau protein to be ubiquitinated and activating downstream breakdown mechanisms that conduct to its degradation throw the proteasome (Salminen et al., 2011).

According to our study, FAD fibroblastic cells had increased HSP levels detected by the proteomics analysis alongside the validation of our results by immunodetection of HSP90 and HSP70 (Figure 9) these data agree with the increase in cellular stress induced by mutant *PS1* reported for somatic cell models (Piccini et al., 2004; Calabrese et al., 2006; Wojsiat et al., 2015). By maintaining oxidative phosphorylation and the functionality of tricarboxylic azide enzymes, increased HSP levels protect against stress induced by intracellular Aβ peptide during AβPP processing (Wilhelmus et al., 2007; Campanella et al., 2018). HSP accumulation could also indicate that other pathogenic factors as well are present in this pathological condition (Adachi et al., 2009; Lyon and Milligan, 2019).

By inducing chaperone expression, *PS1* mutations can also trigger the misfolded protein response (UPR) in the endoplasmic reticulum (ER) and mitochondria due to the accumulation of misfolded proteins including the Aβ peptide and tau protein. Initially, chaperone transcription increases to restore homeostasis but at latter stage, however, chaperone translation is suppressed and its transcription decreases (Koren et al., 2009). It is known that HSP90 regulates tau in coordination with a diverse group of co-chaperones, and the alteration in the levels of heat shock proteins affects therefore the fate of tau and contributes to the onset or the severity of the disease. In this regard, HSP90 inhibition decreases the levels of soluble and insoluble tau protein and of the hyperphosphorylated protein (Shelton et al., 2017).

Hsp70 participates in a variety of folding activities, including the folding and assembly of newly generated proteins, refolding of misfolded and aggregated proteins, membrane translocation of organellar and secretory proteins, and regulatory protein activity regulation. Hsp70 plays a crucial role in the process of chaperone-mediated autophagy (CMA) while in the presence of substrates, hsc70 facilitates substrate binding to LAMP-2A and promotes the formation of the CMA translocation complex; once the substrate has crossed the membrane, Hsp70 actively mediates disassembly of LAMP-2A into monomeric forms (Bandyopadhyay and Cuervo, 2008; Koga and Cuervo, 2011). Continuous assembly and disassembly of the CMA translocation complex guarantees cycles of sequential binding and uptake of substrates by this pathway. There is a strong functional relationship between macroautophagy (non-selective autophagy) and chaperone-mediated autophagy (CMA) pathway by which aggregates of cytosolic proteins are selectively transferred to lysosomes for degradation. Dysregulation of either process CMA, have been observed in fibroblasts from AD patients at the level of macroautophagy (Cuervo and Wong, 2014), and could explain the overexpression of CMA-associated chaperones such as HSP70 reported here (Figure 9) and the high expression of the lysosomal membrane receptor LAMP2 (Figure 2), given the association of these proteins (Wu et al., 2015). Rates of CMA are also directly dependent on the content of LAMP-2A at the lysosomal membrane. Levels of LAMP-2A can be regulated through transcriptional upregulation, as in the case of oxidative stress, or through changes in the degradation rate of LAMP-2A at the lysosomal membrane, as occurs when CMA is upregulated during prolonged starvation (Cuervo and Wong, 2014). Our finding that LAMP2 is overexpressed in fibroblasts from AD patients, may be due to the fact that the group of patients for this study are in the early stages of AD pathology and are still asymptomatic, but the pathological changes/environment could be starting and contributing to the overexpression of LAMP-2A as a compensatory mechanism. Furthermore, as we have mentioned, LAMP2 is significantly elevated in the CSF of AD patients (Armstrong et al., 2014). Mutations in *PS1* lead to increased production of the Aβ peptide, which predisposes to the development of AD, with the consequent activation of macroautophagy, mechanism that promotes Aβ peptide degradation, and alter cell survival. However, autophagy impairment can be relieved by overexpression of HSPs involved in CMA (Morawe et al., 2012).

Our work was focused on peripheral cells, such as fibroblasts, instead of neuronal or brain samples, because the easily availability to obtain these samples, and their ability to model and resemble several mechanisms associated to AD pathology. There is no doubt in the value of studying autopsied brains, brain biopsies, humanized animal models, and extra-neural tissues, and it has allowed for the identification of cellular and molecular changes involved in AD pathophysiology (Joachim et al., 1988; Leinonen et al., 2010; Conejero-Goldberg et al., 2011; LaFerla and Green, 2012; Gomez-Nicola and Boche, 2015; Drummond and Wisniewski, 2017; Hopperton et al., 2018), but all of this approaches require postmortem tissue, invasive way to obtain the samples, ethical limitations and the inconvenient of having to force other species, through overexpression of proteins, to develop something similar to a disease that is exclusive to the human beings. This study, performed on skin fibroblasts, was based on the hypothesis that AD is a systemic disorder that alongside the characteristic brain damage affects other body organs (Francois et al., 2014; Trushina, 2019; Kurakin and Bredesen, 2020). Besides, human skin fibroblasts have been used to elucidate molecular and biochemical mechanisms of pathologies related to metabolic congenital errors linked to neurological diseases and to generate neurons from induced pluripotent stem cells (Auburger et al., 2012; Ambrosi et al., 2014; Bahmad et al., 2017; Pérez et al., 2017; Beh et al., 2020). Therefore, fibroblasts represent a suitable model for neurological genetic diseases with late clinical onset such as FAD because these cells contain the genetic information of the organism from which they originate and have been exposed to the same disease associated environment stress (including epigenetic mechanisms).

Our Western blots showed some variability at the loading controls, this may be due to the fact that there is no perfect housekeeping gene for all tissues and mechanisms analyzed, however, GAPDH has been suggested as the best fit for loading control for stress conditions, such as apoptosis, thus our cell model of patient-derived cells involves stress conditions characteristic of FAD pathology, this is the reason of our selection of GAPDH as the loading control for our Western blots (Ullmannova and Haskovec, 2003). The study of specific target protein expression is often performed by Western blotting, Housekeeping proteins are used as an internal control for protein loading as well as a reference in the Western blotting analysis. Housekeeping genes are considered to be ubiquitously and constitutively expressed in every tissue and produce the minimal essential transcripts necessary for normal cellular function. The most commonly used Housekeeping proteins are β-actin, β-tubulin, and glyceraldehyde 3-phosphate dehydrogenase (GAPDH). However, recent studies have shown significant variation in some Housekeeping genes both at the mRNA and protein levels in various neuropathological events, such as spinal cord injury, Alzheimer’s diseases, and non-neuronal diseases in various tissues. In agreement with Li and Shen (2013), we tested two different Housekeeping genes to normalize our Western blots (Actin and GAPDH), and the less variable was GAPDH.

Molecular, metabolic, and biochemical alterations have been identified in skin fibroblasts from AD patients (Sims et al., 1985, 1987; Peterson and Goldman, 1986; Sorbi et al., 1995; Lee et al., 2010; Pérez et al., 2017), and AβPP processing and Aβ deposition occur in non-neural tissues, supporting that AD is not limited to the brain and thus these facts strengthens the use of peripheral non-neuronal cells for AD research (Puig and Combs, 2013). Accordingly, previous studies performed with peripheral cells from AD patients such as skin fibroblasts, blood lymphoblasts, platelets and oral epidermal cells, show changes in signaling pathways related to cell degeneration, such as the autophagic lysosomal pathway (Martin-Maestro et al., 2017), the proteasome pathway (Munoz et al., 2008), inflammation (Trushina, 2019), lipid metabolism (Francois et al., 2014), cellular stress (Trushina, 2019), cytoskeleton (Takeda et al., 1992), cell survival, and proliferation (Tesco et al., 1992; Hoffmann et al., 2000; Uberti et al., 2002).

In the present study, cultured fibroblasts from FAD patients and healthy individuals showed fusiform morphology and immunoreactivity for Vimentin, a mesenchymal marker (Chang et al., 2002), as well as for the SA100A4 protein (Figure 1), a marker for the fibroblast cell lineage (Strutz et al., 1995; Zhang et al., 2013). Furthermore, we evaluated and characterized the cytogenetic and chromosomal stability of the fibroblasts cell lines used in our study (Supplementary Figure 4) to discard these abnormalities that could happen due to prolonged culture and high-passage number, and none of our cell lines presented any chromosomal instability due to the culture process; only one control line presented a translocation, which is not related to AD (Supplementary Figure 4). It is important to exclude chromosomal abnormalities from the cell lines used, since it could result into histone phosphorylation and cytogenetic instability, which in turn modify somatic cell nuclear transfer, gene expression changes, and cell reprogramming (Allahbakhshian-Farsani et al., 2014).

**FIGURE 10:**
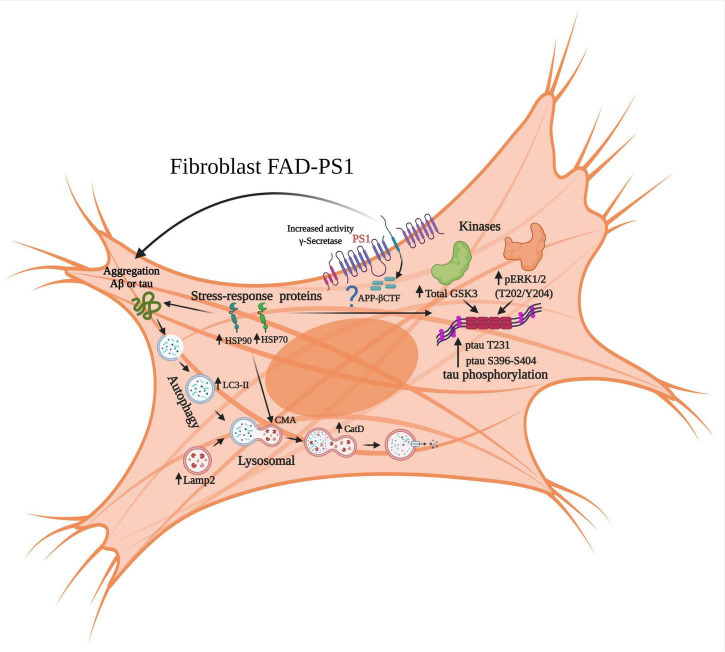
Molecular alterations in signaling pathways associated with neurodegeneration in fibroblasts derived from patients with FAD-*PS1*. The results of gene expression of brain samples from patients with ADF allowed for the identification of changes at the level of the autophagic-lysosomal pathway, as well as of kinases associated with hyperphosphorylation of the protein tau and its pathological phosphorylation. From the proteomic results, it was identified that fibroblasts with *PS1* mutations present increased levels of cellular stress proteins, which could represent a compensation event through chaperone-mediated autophagy and also regulate the aggregation of the Aβ peptide and tau.

In summary, our findings indicate that *PS1* (A246E or M146L) mutated fibroblasts showed alterations at the level of the autophagic-lysosomal pathway, and increased expression or activity of kinases involved in tau phosphorylation. Furthermore, our proteomics study identified increased HSP expression in the total protein extract of FAD individuals, and this finding was validated by immunodetection in all 6 FAD samples (Figures 9, 10). *PS1* mutations in FAD alter different signaling pathways in somatic cells, which could be related to the subsequent appearance of histopathological lesions in the brain, namely amyloid plaques, and neurofibrillary tangles that could be related to the cognitive decline characteristic of this neurodegenerative disease.

Research into the mechanisms by which *PS1* mutations induce the alterations observed in fibroblasts, including other signaling pathways such as calcium regulation, glucose metabolism and oxidative stress identified in our proteomic analysis are warranted.

Clinical trials of about 200 anti-Alzheimer’s disease medication candidates yielded ineffective results, suggesting that the existing AD paradigm may be insufficient, prompting the development of alternative and complementary approaches that allow to generate new hypothesis to approach this complex neurological disease. One important fact is to consider this disease as not exclusive to the CNS, but also a multi-systemic disease, this will facilitate the diagnosis and the generation of new and more accurate therapeutic approaches.

As we know, AD pre-symptomatic stage begins several decades before the clinical phase, therefore it is urgent to develop more efficient diagnosis based on the identification of early biomarkers in peripheral tissues.

Our results therefore support the possibility to identify initial AD pathology associated changes in peripheral cells that provide early evidence of molecular alterations before AD onset that could allow, alongside other clinical criteria, for the early diagnosis of this major devastating neurological disorder that has vast economic and social implications.

## Data Availability Statement

The original contributions presented in this study are included in the article/Supplementary Material, further inquiries can be directed to the corresponding author.

## Author Contributions

M-del-CC-A and GL-T designed the experimental process for the study and wrote and revised the manuscript. GL-T performed all experiments and data analysis presented under the supervision of M-del-CC-A and J-AA-M. M-del-CS-L provided technical assistance in immunofluorescence staining, western blotting, and participated in the review of the work providing intellectual input. JH-D participated in the generation of the 2D gels, data acquisition, analysis, and interpretation of the proteomics studies. J-AA-M and D-EG revised the manuscript. M-del-CC-A conceived the project and supervised its development. All authors approved the final version of the manuscript.

## Conflict of Interest

The authors declare that the research was conducted in the absence of any commercial or financial relationships that could be construed as a potential conflict of interest.

## Publisher’s Note

All claims expressed in this article are solely those of the authors and do not necessarily represent those of their affiliated organizations, or those of the publisher, the editors and the reviewers. Any product that may be evaluated in this article, or claim that may be made by its manufacturer, is not guaranteed or endorsed by the publisher.

## Footnotes

1
https://www.uniprot.org

2
https://string-db.org/

3
http://pantherdb.org/

4
https://www.coriell.org/0/Sections/Search/Sample_Detail.aspx?Ref=AG07629&Product=CC

## Abbreviations

AD: Alzheimer’s disease
FAD: familial Alzheimer’s disease
NA: not affected (control fibroblasts)
Aβ: amyloid-β
AβPP: amyloid β protein precursor
PS1: presenilin-1
PS2: presenilin-2
LC3: microtubule-associated protein light chain 3
LAMP2: lysosome-associated membrane glycoprotein 2
HSP: heat shock protein
GSK3: glycogen synthase kinase-3
S100A4: S100 calcium-binding protein A4.
