# Supplementary material for: Patient-Derived Fibroblasts With Presenilin-1 Mutations, That Model Aspects of Alzheimer’s Disease Pathology, Constitute a Potential Object for Early Diagnosis
Source: Front Aging Neurosci. 2022 Jul 1;14:921573. doi: 10.3389/fnagi.2022.921573 (PMC9283986; doi:10.3389/fnagi.2022.921573)
Supplement: Supplementary file 1 [file Data_Sheet_1.DOCX]

Supplementary Material

# Supplementary Figures


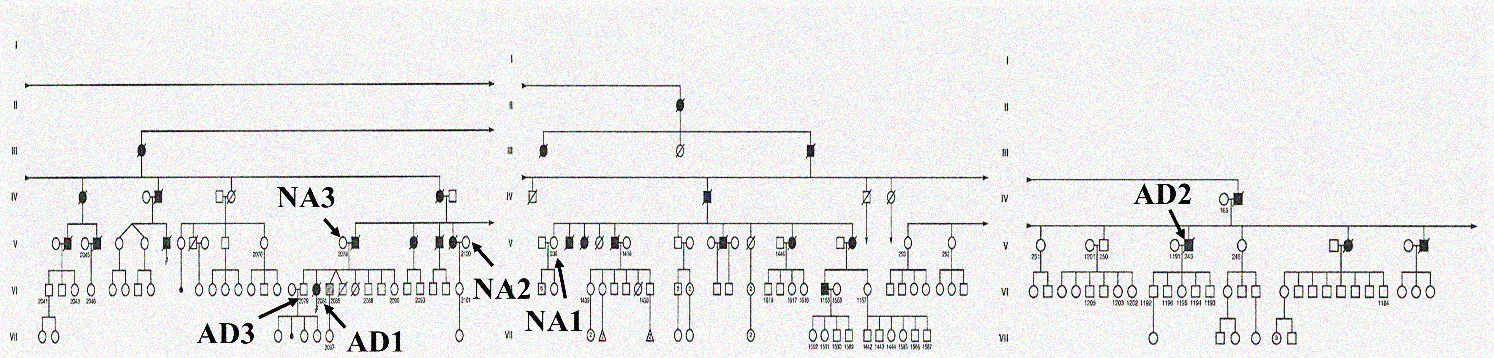


**Supplementary figure 1. Identification of individuals with familial AD (*PS1*-M146L) and controls in the genealogical tree of the Italian family.** The fibroblast cell lines were reported as AD1, AD2, AD3, NA1, NA2 and NA3 (AD: Alzheimer's disease; NA: Not affected with FAD). The image was modified from Fonci et al. (1985).


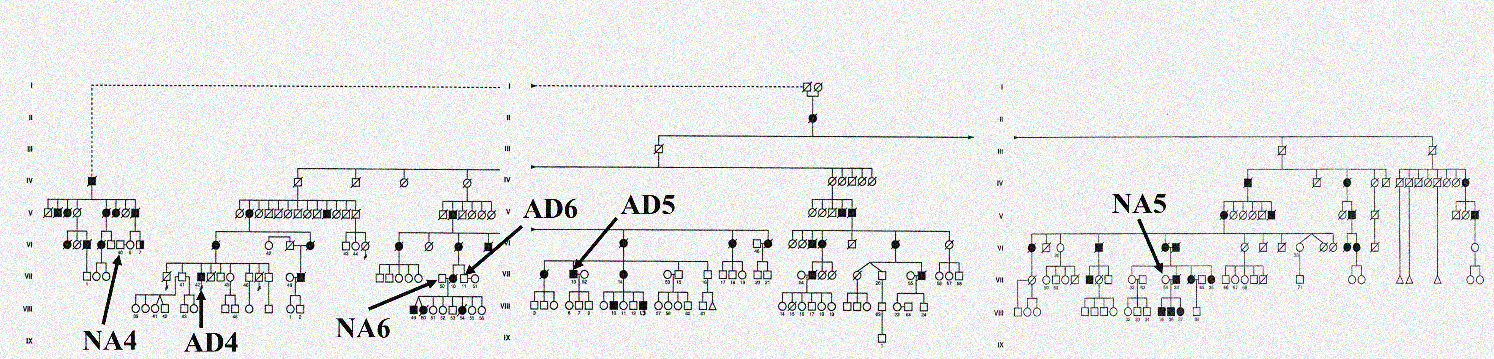


**Supplementary figure 2. Identification of individuals with familial AD (*PS1*-A246E) and controls in the genealogical tree of the Canadian family.** The fibroblast cell lines were reported as AD4, AD5, AD6, NA4, NA5 and NA6 (AD: Alzheimer's disease; NA: Not affected with FAD). The image was modified from Nee et al. (1983).


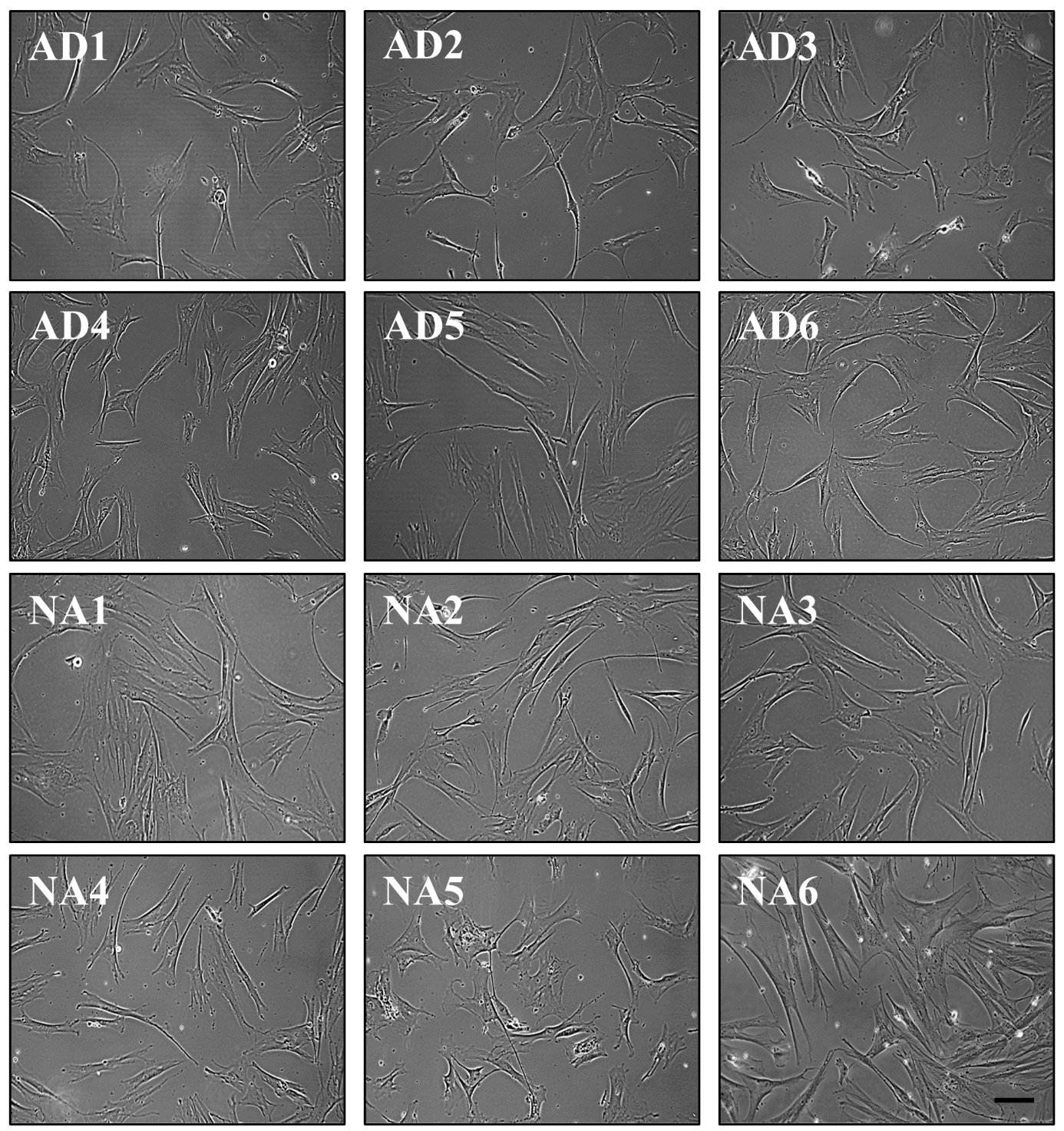


**Supplementary figure 3. Culture of FAD fibroblasts and controls.** Cells were grown in Minimal Essential Medium with Earl salts supplemented with 15% non-inactivated FBS. All 12 cell lines (6 FAD and 6 controls) show spindle and lamellar morphologies. Images were obtained from culture passages 10 to 20, with a 20X objective. The scale bars correspond to 50 µm.


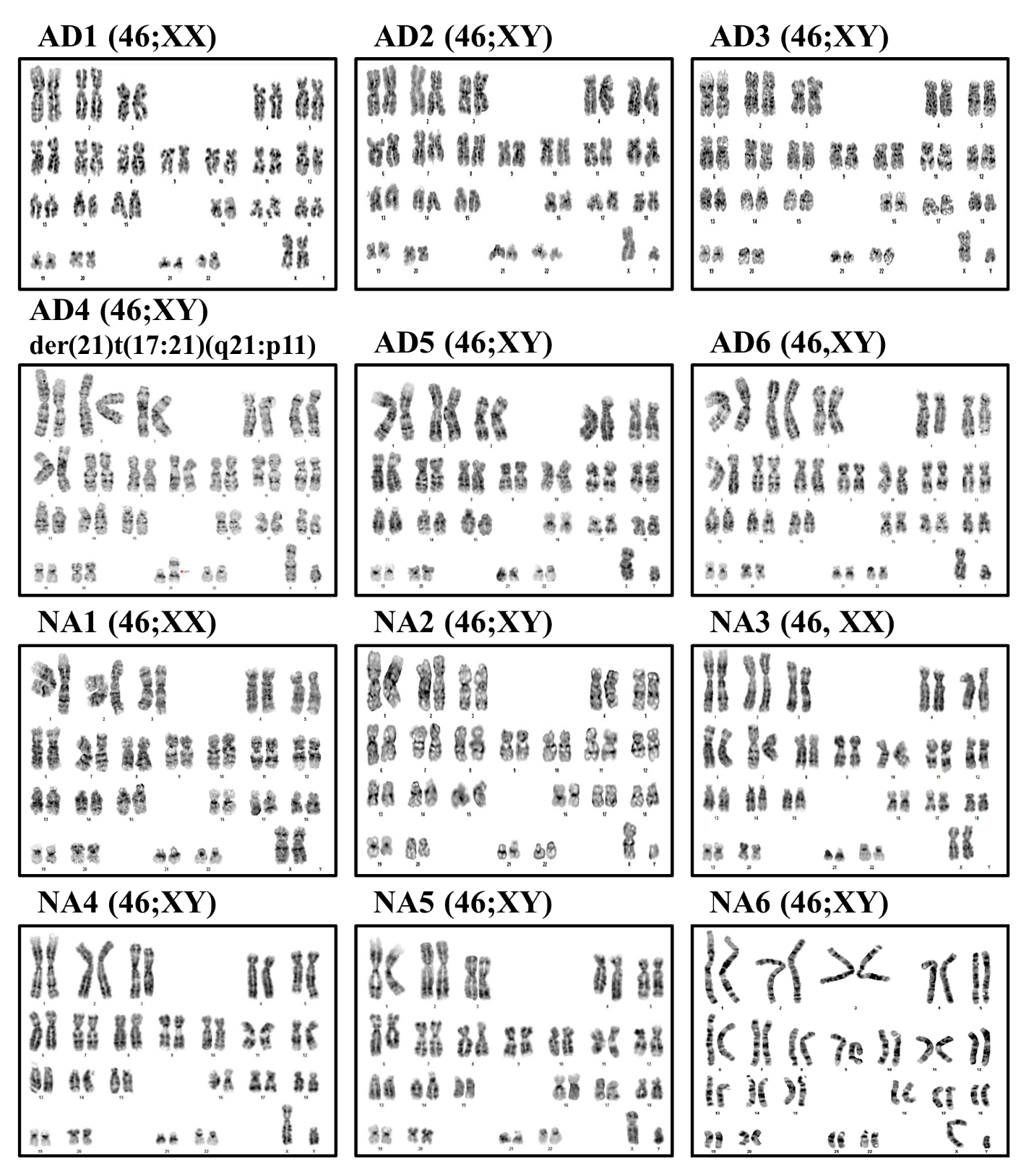


**Supplementary figure 4. Karyotypes of fibroblasts from FAD individuals and control subjects.** Nine male (46; XY) and 3 female (46; XX) karyotypes, with a normal chromosomal number, were identified. The cell line AD4 has a translocation (17:21). For G-band karyotypes, images are representative of 17-20 metaphases. Images were acquired with a 100X objective.
